# Slow-Growing Pancreatic Smooth Muscle Tumor Treated with Partial Pancreatectomy: A Case Report

**DOI:** 10.70352/scrj.cr.25-0305

**Published:** 2025-10-01

**Authors:** Yoshiki Murase, Masayasu Aikawa, Yu Miyama, Yuichiro Watanabe, Kenichiro Takase, Yukihiro Watanabe, Hiroaki Ono, Katsuya Okada, Kojun Okamoto, Isamu Koyama

**Affiliations:** 1 Department of Hepato-Biliary-Pancreatic Surgery, Saitama Medical University International Medical Center, Hidaka, Saitama, Japan; 2 Department of Pathology, Saitama Medical University International Medical Center, Hidaka, Saitama, Japan

**Keywords:** leiomyoma, pancreatectomy, pancreatic neoplasms, smooth muscle tumor

## Abstract

**INTRODUCTION:**

Smooth muscle tumors, such as leiomyoma or leiomyosarcoma, are mesenchymal tumors that originate from smooth muscle cells. Smooth muscle tumors in the abdominal cavity often originate from the uterus, gastrointestinal wall, and inferior vena cava, but rarely originate from the pancreas. We present a rare case of a pancreatic leiomyoma that was successfully treated via partial pancreatectomy.

**CASE PRESENTATION:**

A 74-year-old man with a pancreatic head tumor was referred to our hospital. Imaging revealed a heterogeneous 61 × 45 mm mass with a contrast effect in the pancreatic head. Distant metastases were not detected. The tumor was diagnosed by endoscopic ultrasonography-guided fine-needle aspiration as a leiomyoma, and follow-up was planned. Five years after the initial visit, the patient developed abdominal tension, and a CT scan revealed that the tumor had grown to 80 × 60 mm. It was decided that the tumor required surgical treatment comprising pancreaticoduodenectomy. However, the intraoperative findings revealed that the tumor was able to be moved freely but was unable to be dissected from a part of the pancreatic head. Therefore, partial pancreatectomy was performed. Histopathology showed spindle-shaped tumor cells with mild atypia. Pancreatic tissue was present at the tumor margins, and the tumor was adjacent to the vasculature. Based on the clinical and pathological findings, the tumor was diagnosed as a smooth muscle tumor originating from the pancreas. The patient was discharged without complications on POD 8 and has now been doing well without recurrence for more than 6 months after surgery.

**CONCLUSIONS:**

A very rare slow-growing pancreatic smooth muscle tumor was removed via partial pancreatectomy after the patient developed abdominal symptoms after a 5-year observation period.

## Abbreviation

EUS-FNA
: endoscopic ultrasonography-guided fine-needle aspiration

## INTRODUCTION

Most pancreatic tumors are ductal adenocarcinomas arising from the epithelium of the pancreatic duct, while mesenchymal pancreatic tumors are rare.^1–3)^ Smooth muscle tumors are a type of mesenchymal tumor that originates from smooth muscle cells and are generally classified as benign leiomyomas and malignant leiomyosarcoma.^4)^ Leiomyomas in the abdominal cavity often originate from smooth muscles such as the uterus, gastrointestinal wall, and inferior vena cava, but rarely originate from the pancreas.^5)^ Herein, we present a case in which a slow-growing pancreatic smooth muscle tumor thought to be a leiomyoma required differentiation from a leiomyosarcoma and was successfully treated by partial pancreatectomy.

## CASE PRESENTATION

**Fig. 1:**
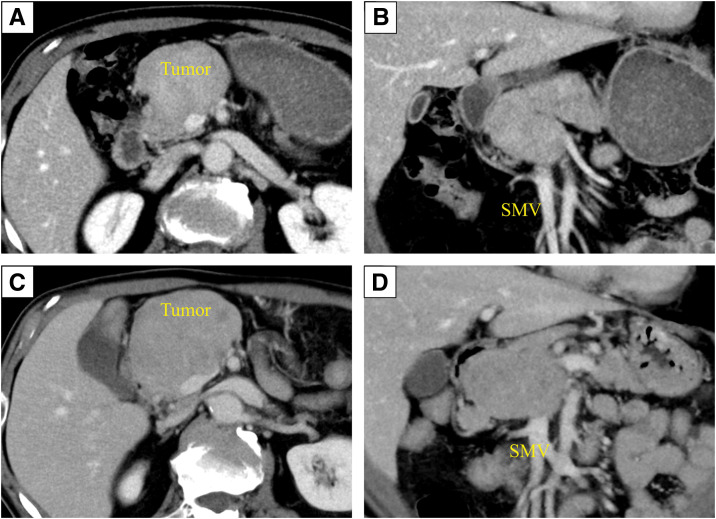
Abdominal CT imaging at the initial diagnosis and after 5 years. (**A**, **B**) CT imaging at the initial diagnosis reveals a 61 × 45 mm heterogeneous enhanced tumor in the pancreatic head that does not invade the surrounding tissue but is in contact with the SMV. (**C**, **D**) On imaging obtained 5 years after the initial diagnosis, the tumor has grown to 80 × 60 mm without invasion into the surrounding tissue. SMV, superior mesenteric vein

**Fig. 2:**
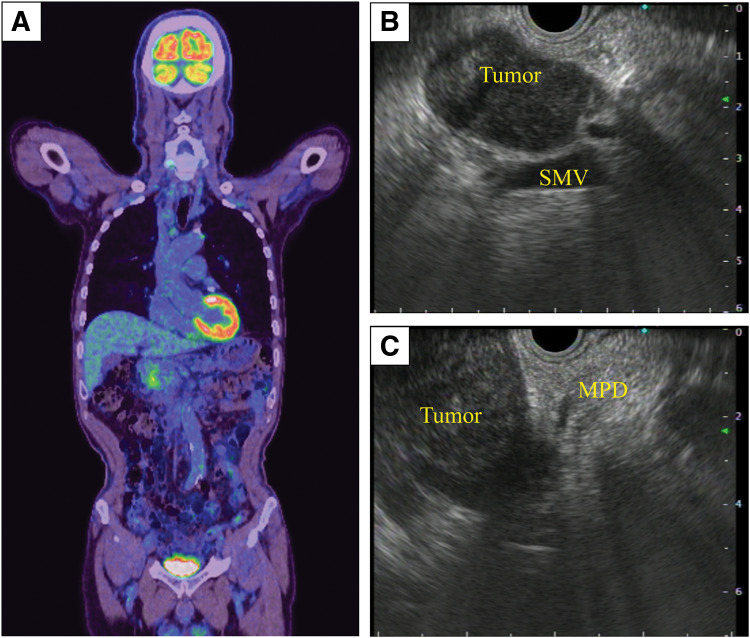
PET-CT and EUS imaging. (**A**) PET-CT at the initial diagnosis shows increased metabolic activity in the pancreatic head. (**B**, **C**) EUS imaging at the initial diagnosis shows that the tumor is contiguous with the pancreas, without invasion into the SMV. EUS, endoscopic ultrasonography; MPD, main pancreatic duct; SMV, superior mesenteric vein

A 74-year-old man was referred to our hospital because a mass in the pancreatic head was detected during a medical checkup. His medical history comprised diabetes mellitus and angina pectoris. Laboratory data showed no increases in the concentrations of the tumor markers carcinoembryonic antigen (1.7 ng/mL) and carbohydrate antigen 19-9 (2.8 U/mL). CT showed a heterogeneous 61 × 45 mm mass with a contrast effect in the pancreatic head. The tumor appeared to be contiguous with the pancreas, but there was no invasion into the surrounding tissue (**Fig. 1A**–**1D**). PET-CT showed increased metabolic activity in the pancreatic head (maximum standardized uptake value for the tumor was 3.1), but no evidence of metastases in other organs (**Fig. 2A**). An endoscopic ultrasonography-guided fine-needle aspiration (EUS-FNA) of the pancreatic head tumor was performed (**Fig. 2B** and **2C**). Histological examination showed a proliferation of spindle-shaped cells. On immunohistochemical evaluation, the tumor cells were positive for desmin and negative for c-KIT, DOG-1, and S100. The Ki-67 labeling index was 2%. Based on these findings, we decided to monitor the patient without intervention because the tumor was most likely a benign leiomyoma.

Five years after the initial visit, the patient developed abdominal tension, and a CT scan revealed that the tumor had grown to 80 × 60 mm (**Fig. 1C** and **1D**). Therefore, it was decided that the tumor required surgical treatment comprising pancreaticoduodenectomy.

**Fig. 3:**
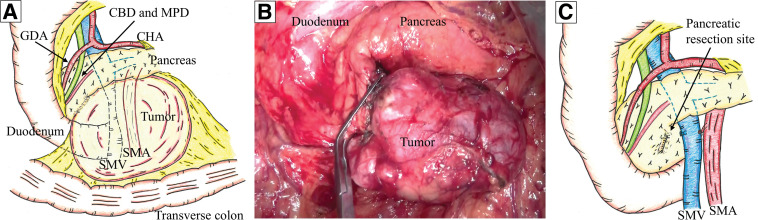
Intraoperative findings and schematic illustrations. (**A**) Preoperative schematic illustration shows that the tumor originates from a part of the pancreatic head. The tumor showed no invasion of surrounding organs, the CBD, or the MPD, although the SMV is compressed by the tumor. (**B**) Intraoperative findings show that the tumor moves freely but is unable to be dissected from a part of the pancreatic head. (**C**) The schematic illustration after tumor resection shows that a partial pancreatectomy was performed on the pancreatic head. CBD, common bile duct; CHA, common hepatic artery; GDA, gastroduodenal artery; MPD, main pancreatic duct; SMA, superior mesenteric artery; SMV, superior mesenteric vein

Intraoperative findings revealed that the tumor was able to be moved freely but was unable to be dissected from a part of the pancreatic head. There was no enlargement of the lymph nodes surrounding the tumor that would have been suggestive of metastasis. Therefore, we performed a local resection of the pancreatic head as partial pancreatectomy (**Fig. 3A**–**3C**). The total duration of surgery was 153 min, and there was minimal blood loss. The patient was discharged without complications on POD 8.

**Fig. 4:**
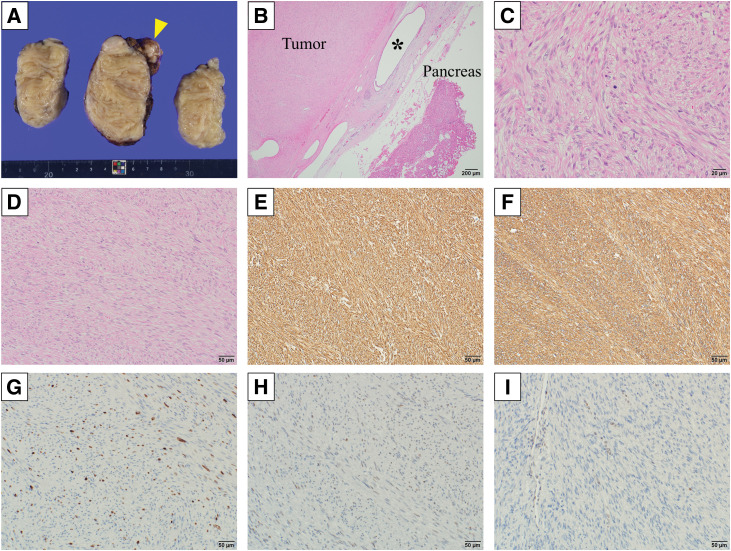
Surgical specimen and pathological findings. (**A**) Surgical specimen consisting of the tumor and part of the pancreas (yellow arrow). (**B**, **C**) Micrographic imaging of hematoxylin- and eosin-stained specimens of the tumor and pancreatic tissue. The pathological findings show a hypercellular tumor that is continuous with the intrapancreatic vasculature, and the pancreatic tissue with a section of the pancreatic duct (*). The hypercellular stromal tumor comprises spindle cells with fibrillar cytoplasm. Mitosis is confirmed in 1–5 cells per 50 high-powered fields. (**D**) Hematoxylin and eosin staining shows tumor cells with mild atypia forming intersecting fascicles. The tumor cells are positive for (**E**) h-caldesmon and (**F**) alpha-smooth muscle actin. (**G**) The Ki-67 labeling index of the tumor is 12%. (**H**) The tumor focally expresses p53, which suggests the wild type of *TP53*. (**I**) The Rb1 expression is lost in tumor cells but retained in the endothelial cells used as a control.

The resected specimen was an 80 × 65 × 50 mm, white, well-demarcated fibrous tumor with a part of the pancreas (**Fig. 4A**). Histopathological findings revealed spindle-shaped tumor cells with mild atypia forming intersecting fascicles. Pancreatic tissue was present at the tumor margins, and the pancreatic vasculature was adjacent to the tumor (**Fig. 4B**). The tumor exibihted a few mitotic figures (0–1 per mm^2^) and no tumor cell necrosis (**Fig. 4C**). Immunohistochemistry showed strong positive staining for h-caldesmon and alpha-smooth muscle actin, with negativity for desmin, MDM2, and CDK4 (**Fig. 4D**–**4F**). The Ki-67 labeling index was 12% (**Fig. 4G**). The p53 expression of the tumor was focal, suggesting the wild type of *TP53* (**Fig. 4H**). Rb1 expression was lost (**Fig. 4I**). These findings were consistent with a smooth muscle tumor. Based on the clinical and pathological findings, the tumor was diagnosed as a smooth muscle tumor originating from the pancreas.

The patient has been doing well without recurrence for more than 6 months after the surgical treatment.

## DISCUSSION

Pancreatic smooth muscle tumor is a very rare type of mesenchymal tumor. Herein, we reported a patient with a pancreatic smooth muscle tumor suggested to be a leiomyoma that was removed via partial pancreatectomy after being observed without intervention for 5 years.

A literature search revealed that there have been less than 10 reported cases of pancreatic leiomyoma,^5–11)^ each with very interesting and varied clinical presentations and outcomes in **Table 1**. Previous reports of pancreatic leiomyoma involved 4 men and 3 women with a median age of 62 years (range 21–75 years). Three of the patients each presented with a symptom comprising dyspeptic symptoms, abdominal pain, and obstructive jaundice, respectively.^5,7,10)^ The tumor originated from the pancreatic head in all cases, and the median tumor diameter was 4 cm (range 2.5–14 cm). Two patients received follow-up only, 2 underwent partial pancreatectomy, and 3 underwent pancreaticoduodenectomy. There were 3 cases in which the diagnosis of pancreatic leiomyoma was made preoperatively or during follow-up.^5,7,11)^ The present case had a similar clinical background, including tumor size and location, to the previously reported cases, but this was the 1st case in which the patient was diagnosed preoperatively with pancreatic leiomyoma, followed up for 5 years, and then underwent surgery after symptoms developed. Therefore, the present case may show one of the surgical indications for pancreatic leiomyoma, as no previous reports have considered a surgical indication for this disease. At the time of the initial diagnosis, the present patient had no symptoms and the tumor was discovered incidentally. After careful examination, the tumor was diagnosed as leiomyoma, suggesting a benign tumor. Considering the findings of PET-CT, tumor size, or the possibility of sampling error in EUS-FNA, surgical resection was also considered at the time of diagnosis. However, since the patient requested observation and there have been previous case reports in which only observation was performed for pancreatic leiomyomas,^7,11)^ we initially opted to perform follow-up only. The tumor size had not changed at the 1-year follow-up. However, at the 5-year follow-up, despite no changes in tumor size or symptoms up to that point, the tumor size had increased and abdominal symptoms had appeared. In addition to these points, the patient expressed a desire to undergo surgery. When the patient decided to undergo surgery, he was 79 years old. In general, since pancreatic resection in elderly patients is thought to affect postoperative outcomes, the decision of the surgery should be made after considering the patient’s overall condition. The patient’s performance status was good, and no complications that would prevent surgery were found in the preoperative examination. Therefore, the decision was made to perform surgery. These findings are generally considered indications for tumor resection, and it seems that these indications should also apply to leiomyomas, even though these are benign tumors.

**Table 1:** Previous reports of pancreatic leiomyoma

Author	Year	Gender	Age	Presentation	Tumor location	Tumor size (cm)	Preoperative diagnosis	Treatment	Long-term outcome
Nakamura et al.^8)^	2000	Female	72	Incidental findings	Head	5.5	Islet-cell tumor	Enucleation	No recurrence at 1 year
Wisniewski et al.^6)^	2006	Male	52	Incidental findings	Head	2.5	Benign tumor	PD	No recurrence at 1 year
Sato et al. ^11)^	2012	Female	62	Incidental findings	Head	3.5	Leiomyoma	Observation	N.A.
Kant et al.^7)^	2021	Male	75	Dyspeptic symptoms	Head	4	Leiomyoma	Observation	Change to malignancy at 13 years
Luo et al.^9)^	2022	Male	74	Incidental findings	Head	4	PNET or cystadenoma	Partial pancreatectomy	No recurrence at 6 months
Petchpiboolthai et al.^10)^	2023	Female	31	Abdominal pain	Head	14	GIST or SPN	PD	No recurrence at 2 months
Balani et al.^5)^	2024	Male	21	Obstructive jaundice	Head	7	Leiomyoma	PD	No recurrence at 1 year
Present case	2025	Male	74	Medical checkup	Head	8	Leiomyoma	Partial pancreatectomy	No recurrence at 6 months

GIST, gastrointestinal stromal tumor; N.A., not available; PD, pancreaticoduodenectomy; PNET, pancreatic neuroendocrine tumor; SPN, solid pseudopapillary neoplasm

The origin of the tumor in the present case was thought to be the pancreas for 2 reasons. The 1st was that the tumor could be dissected from the surrounding tissue but not from the pancreas, and the 2nd was that the tumor and pancreas were pathologically contiguous.^8,10)^ In the present case, the tumor was adherent only to the pancreatic head, while the surrounding tissue was easily dissected. Moreover, histopathology showed that there was pancreatic tissue at the tumor margins and the tumor was continuous with the vasculature adjacent to the pancreas. Therefore, we determined that the tumor originated in the pancreas.

One of the diseases from which leiomyoma must be differentiated is leiomyosarcoma. Leiomyosarcoma of the pancreas is a malignant pancreatic mesenchymal tumor that is reported to be more common than leiomyoma.^4)^ It is very important to distinguish between leiomyoma and leiomyosarcoma because the prognoses of the diseases are different. In pathological differences between leiomyoma and leiomyosarcoma, leiomyosarcoma was reported to have strong nuclear atypia and high mitotic activity. Sometimes, necrosis, pleomorphism, or focal myxoid change were observed in leiomyosarcoma.^12)^ However, due to the rarity of pancreatic mesenchymal tumors, there are no previous reports of a method to differentiate pancreatic leiomyoma from pancreatic leiomyosarcoma. Therefore, we were cautious in our attempts to differentiate between these 2 pancreatic tumors. As the tumor in the present case had grown relatively slowly over a 5-year period and preoperative imaging and intraoperative findings showed no invasion into the surrounding tissues, the tumor was considered clinically negative for malignancy. In addition, the lack of nuclear atypia, absence of necrosis, wild strain of p53, and not extremely high Ki-67 index suggested that the tumor was pathologically benign. However, the presence of a few mitotic figures and the loss of Rb indicated that the tumor might not be benign. Kant et al. reported a pancreatic leiomyoma that transformed into a leiomyosarcoma during a 13-year period.^7)^ Although we could not rule out the possibility that the present case also involved the morphological transformation of a leiomyoma into a leiomyosarcoma, we were unable to prove this. Finally, after considering the clinical and pathological findings, we determined that the tumor was a smooth muscle tumor that required careful follow-up.

## CONCLUSIONS

A slow-growing pancreatic smooth muscle tumor was observed for 5 years before being removed via partial pancreatectomy after the onset of abdominal symptoms. Even if the tumor was diagnosed as benign, when the signs of malignancy, such as tumor growth, the appearance of symptoms, or changes in imaging findings, were recognized, surgical resection should be considered promptly. This treatment strategy may be an excellent option for pancreatic smooth muscle tumors suggested to be leiomyomas, as there are few reported cases of the treatment of pancreatic leiomyoma.

## DECLARATIONS

### Availability of data and materials

All data generated during this report are included in the published article.
